# *Fusobacterium* Chorioamnionitis: Report of Two Cases in Preterm Labor With Intact Amniotic Membranes

**DOI:** 10.1155/S1064744996000464

**Published:** 1996

**Authors:** Allahyar Jazayeri, Michael E. Lantz, William F. O'Brien

**Affiliations:** 1 Department of Obstetrics and Gynecology University of South Florida College of Medicine Tampa FL USA; 2 Suite 500 Harbourside Medical Tower 4 Columbia Drive Tampa FL 33606 USA

## Abstract

*Background:* Preterm labor (PTL) in women with intact membranes may be caused by developing chorioamnionitis. *Fusobacterium* displays the ability to cause chorioamnionitis in the presence of intact amniotic membrane.

*Case:* We report 2 patients with severe *Fusobacterium* chorioamnionitis which resulted in premature termination of pregnancy. Both patients presented with PTL and intact membranes. Neither initially appeared acutely ill. Despite the benign appearance, one woman rapidly deteriorated, requiring ventilation, pressor support, and surgical evacuation of the uterus.

*Conclusion:* We feel that a Gram's Stain and proper collection of anaerobic cultures at the time of amniocentesis should be part of the evaluation of every patient with suspected chorioamnionitis.


Infectious Diseases in Obstetrics and Gynecology 4:243–246 (1996)
© 1997 Wiley-Liss, Inc.

### *Fusobacterium* Chorioamnionitis: Report of Two Cases in Preterm Labor With Intact Amniotic Membranes

Allahyar Jazayeri, Michael E. Lantz, and William F. O'Brien

*Department of Obstetrics and Gynecology, University of South Florida College of Medicine, Tampa, FL*

ABSTRACT

***Background:*** **Preterm labor (PTL) in women with intact membranes may be caused by developing chorioamnionitis. *Fusobacterium* displays the ability to cause chorioamnionitis in the presence of intact amniotic membrane.**

***Case:*** **We report 2 patients with severe *Fusobacterium* chorioamnionitis which resulted in premature termination of pregnancy. Both patients presented with PTL and intact membranes. Neither initially appeared acutely ill. Despite the benign appearance, one woman rapidly deteriorated, requiring ventilation, pressor support, and surgical evacuation of the uterus.**

***Conclusion:*** **We feel that a Gram's stain and proper collection of anaerobic cultures at the time of amniocentesis should be part of the evaluation of every patient with suspected chorioamnionitis.** © 1997 Wiley-Liss, Inc.

KEY WORDS

Intraamniotic infection, anaerobic infection, anaerobic cultures, sepsis, twin gestation

The association between preterm labor (PTL) and intraamniotic infection is well known. However, a search for obvious chorioamnionitis in a patient presenting with PTL often reveals no identifiable infection.[1] Even though amniocentesis is frequently used to detect intraamniotic infections in PTL,[2–4] cultures are not always collected under anaerobic conditions.[5] Although anaerobic infection as a cause for PTL was reported as early as 1966,[6,7] the importance of *Fusobacterium* as an etiologic pathogen for PTL has only been recognized in the past decade.[8–10] This organism displays the unique ability to cause chorioamnionitis in the presence of intact amniotic membranes.[2,11,12]

We present 2 cases in which *Fusobacterium* was found in the amniotic fluid of patients presenting with preterm contractions and intact membranes.

### CASE REPORTS

CM was a 25-year-old primigravida who presented at 23 weeks estimated gestational age with a twin gestation. She complained of contractions every 5 min but denied any leakage of fluid or vaginal discharge. The patient had had an uneventful prenatal course with normal fetal movement up until the time of presentation. Moreover, she had no significant medical history.

Upon her presentation, she had a temperature of 37.5°C, a pulse rate of 111 beats/min (bpm), a respiratory rate of 24 (breaths/min), and a blood pressure of 126/72 mmHg. The lung fields were clear to auscultation, and the uterus was non-tender. The vaginal examination revealed no pooling of fluid in the vaginal vault. Nitrazine and fern testing were negative but clue cells were present.

Address correspondence/reprint requests to Dr. Allahyar Jazayeri, Suite 500, Harbourside Medical Tower, 4 Columbia Drive, Tampa, FL 33606.

Received April 24, 1996
Accepted August 2, 1996

The cervix was 1 cm dilated and 50% effaced. The vertex of twin A was blottable. An ultrasound examination confirmed the gestational age, a dividing membrane, and normal amniotic-fluid volume. Her urinalysis was normal, WBC count was $13.6 \times 10^9$/l, and hematocrit was 30.2%. There were uterine contractions every 5–7 min. The fetal heart rate was in the 160-bpm range without decelerations.

An amniocentesis of both amniotic sacs revealing clear amniotic fluid was sent to the laboratory immediately. The Gram's stains of both amniotic fluids revealed gram-negative rods (twin A with many gram-negative rods and twin B with few gram-negative rods) and many WBCs. The Gram's stains were performed with commercially available reagents using a kit from DIFCO (Detroit, MI). The glucose levels obtained from the 2 sacs were 2 and 6 mg/dl, respectively. The diagnosis of intraamniotic infection was made and the patient was started on oxytocin, ampicillin, gentamicin, and clindamycin.

Approximately 4 h after her presentation, the patient became acutely short of breath. Her temperature was 39.3°C, pulse 120 bpm, blood pressure 102/50 mmHg, and oxygen saturation 78%. The uterus was markedly tender, with contractions every 3 min, and the cervix was 3 cm dilated. The patient subsequently became more short of breath, with an oxygen saturation of 60%, and required intubation. She was immediately taken to the operating room for evacuation of the uterus.

After the procedure, a Swan-Ganz catheter was placed. Her initial pulmonary artery pressure was 57/39 mmHg, pulmonary capillary wedge pressure 7 mmHg, cardiac output 5.92 l/min, and systemic vascular resistance 446 dyn · s/cm$^5$. A chest X-ray showed extensive bilateral pulmonary infiltrates. The WBC count was $13.9 \times 10^9$/l, hematocrit 26.3%, and platelet count $189 \times 10^{12}$/l. The serum electrolytes and coagulation parameters were within normal limits.

The patient was administered supportive therapy including vasopressors. She developed a 30% pneumothorax of the right lung which required chest tube placement. She was afebrile after 48 h and extubated on postoperative day 4. The remainder of her hospital course was uneventful and she was discharged on postoperative day 7.

The amniotic-fluid culture from both twins grew *F. nucleatum* after 4 days. A cervical culture showed light growth of urogenital flora and heavy growth of *Gardnerella vaginalis*. The fetal autopsies were normal except for severe, acute chorioamnionitis and funisitis (twin A had severe acute chorioamnionitis and twin B had mild-to-moderate acute chorioamnionitis). Both placental cultures were positive for *F. nucleatum* after 5 days. The placenta from twin A also grew *Peptostreptococcus anaerobius*, while the placenta from twin B grew *Escherichia coli*. Maternal blood, urine, and sputum cultures as well as cervical cultures for gonococcus and chlamydia were negative.

MB was a 42-year-old $G_3P_{1011}$ who presented at 24 weeks gestation with a green vaginal discharge. She denied contractions, leakage of fluid, and vaginal bleeding. Her prenatal course had been uncomplicated. She had undergone a genetic amniocentesis 17 days previously which revealed a normal male karyotype. Her medical history was significant for a laparoscopy for pelvic pain in 1992, a gonococcal infection in 1971, and intravenous (IV) heroin use. She reported having been free of heroin use for the last 10 years and in a methadone maintenance program. She denied leakage of amniotic fluid after her amniocentesis.

Her physical examination revealed a temperature of 38.5°C, pulse 77 bpm, respiratory rate 18/min, and blood pressure 115/57 mmHg. The uterus was not tender. The vaginal vault showed no pooling. The green vaginal discharge was Nitrazine positive without evidence of ferning. The cervix was 1 cm dilated and uneffaced, with a blottable presenting part. There were contractions every 5 min and a fetal heart rate of 160–180 bpm.

An ultrasound examination confirmed the gestational age. A single fetus was in a breech presentation with an estimated fetal weight of 575 g. The amniotic fluid index was 11.7 cm.

Over the next 6 h, the patient continued to have contractions and her cervix changed to 2 cm, with 50% effacement at a −3 station. $MgSO_4$ tocolysis was initiated, an amniocentesis was performed, and prophylaxis with ampicillin was started. Clue cells were noted in the vaginal discharge. The WBC count was $14.7 \times 10^9$/l with 5% bands, the hematocrit 36.4%, and the platelet count $244 \times 10^{12}$/l. A urinalysis was negative.

The amniocentesis yielded approximately 20 ml of cloudy, yellow fluid. The gram's stain showed sheets of gram-negative rods and many WBCs. The glucose concentration was 6 mg/dl. The diagnosis of intraamniotic infection was made. $MgSO_4$ tocolysis was stopped, and the patient was taken to the operating room within 1 h of the amniocentesis. The patient was delivered by a classical cesarean of a live male infant weighing 545 g. Gentamicin and clindamycin were started at the time of the cesarean. After having an uneventful postoperative course, she was discharged home on postoperative day 4.

The cervical cultures obtained on the patient's presentation subsequently showed rare growth of urogenital flora and light growth of *Gardnerella vaginalis.* The amniotic-fluid cultures grew *F. nucleatum.* The placental cultures were negative as well as all other cultures. The pathologic examination of the placenta showed severe, acute chorioamnionitis. The infant's course was complicated by respiratory distress syndrome, cardiac arrest, hyaline-membrane disease, a grade-IV intraventricular hemorrhage, and marked dilation of the lateral ventricles. He was eventually discharged home with guarded prognosis on oxygen and apnea monitoring for apnea of prematurity.

### DISCUSSION

*Fusobacterium* species are anaerobic gram-negative rods that are differentiated from the family Bacteroidaceae by their morphology, antibiotic sensitivity, and production of specific organic acids.[10] On a Gram's stain, *Fusobacterium* species appear pleomorphic and filamentous.[9] They are frequent oral pathogens, but infrequently pathogens in pelvic infections. Although the incidence of *Fusobacterium* in cervical cultures of pregnant women in the early third trimester is low (2.9%),[13] in cases of PTL in which the amniotic-fluid cultures are positive, a significant number (30.4%) will grow *Fusobacterium.*[10]

One of the major concerns in identifying *Fusobacterium* as a pathogen is the sensitivity of the culture technique. Many anaerobic bacteria are extremely sensitive to air and unable to tolerate any exposure. Chow et al.[14] have described specific guidelines for the collection and processing of anaerobic cultures to preserve the ability of these organisms to grow in vitro. Gravett et al.[15] used a commercially available transport system for the collection and processing of anaerobic cultures of amniotic fluid. In our institution, anerobic cultures of amniotic fluid are collected and placed in a commercially available transport tube (BMI-S Borth Becton Dickson, BBL, Cockeysville, MD). Subsequently, the amniotic fluid is cultured on CDC and K-V plates (Becton Dickson, BBL) and in BHI-S broth media.

The case reports of *Fusobacterium* causing intraamniotic infection with intact membranes are few. Chaim and Mazor,[16] in a review of intraamniotic infection with *Fusobacterium,* cited an incidence of *Fusobacterium* in culture-positive amniotic fluid that ranged from 15.4 to 100%, averaging 28.3%. In premature rupture of the membranes, a significantly lower percentage of positive amniotic-fluid cultures grew *Fusobacterium* (9.9%). These authors speculated that these data indicate the importance of *Fusobacterium* as an etiologic factor in PTL with intact membranes.

We report 2 cases of *Fusobacterium* chorioamnionitis with intact membranes, 1 of which resulted in significant maternal morbidity. On initial presentation, neither patient appeared critically ill despite severe chorioamnionitis. In the patient with a twin gestation, acute deterioration resulted in respiratory failure and septic shock, requiring surgical evacuation of the uterus. With the rapid progression of this infection to septic shock, the cardiovascular changes associated with a twin gestation may have been partially responsible. Easterling and Garite,[10] however, have reported a similar course in a woman with a singleton gestation complicated by *Fusobacterium* chorioamnionitis.

With the potential serious complications, of *Fusobacterium* infection, the index of suspicion for chorioamnionitis with this organism should be elevated in women with intact membranes. Therefore, we recommend that amniotic fluid be collected for microbial culture under anaerobic conditions and processed accordingly. A Gram's stain of this fluid may identify *Fusobacterium* as the causative agent in suspected chorioamnionitis and guide antibiotic therapy, which should be initiated as soon as the diagnosis of chorioamnionitis is made. Prompt diagnosis and therapy are particularly important since the clinical course of *Fusobacterium* infection can progress rapidly to sepsis with significant maternal and fetal morbidity and even mortality. We, therefore, strongly advocate the early initiation of broad-spectrum antibiotics in all cases in which *Fusobacterium* chorioamnionitis is suspected.

### REFERENCES

1. Wallace RL, Herrick CN: Amniocentesis in the evaluation of premature labor. Obstet Gynecol 57:483–486, 1981.
2. Weible DR, Randall HW Jr: Evaluation of amniotic fluid in preterm labor with intact membranes. J Reprod Med 30:777–780, 1985.
3. Leigh J, Garite TJ: Amniocentesis and the management of premature labor. Obstet Gynecol 67:500–506, 1986.
4. Skoll MA, Moretti ML, Sibai BM: The incidence of positive amniotic fluid cultures in patients in preterm labor with intact membranes. Am J Obstet Gynecol 161:813–816, 1989.
5. Pankuch GA, Appelbaum PC, Lorenz RP, et al.: Placental microbiology and histology and the pathogenesis of chorioamnionitis. Obstet Gynecol 64:802–806, 1984.
6. Pearson HE, Anderson GV: Perinatal infections associated with *Bacteroides* infections. Obstet Gynecol 30:486–492, 1967.
7. Townsend L, Aickin DR, Fraillon JMG: Spontaneous premature rupture of membranes. Aust NZ J Obstet Gynecol 6:226–235, 1966.
8. Gibbs RS, Blanco JD, St Clair PJ, et al: Quantitative bacteriology of amniotic fluid from women with clinical intraamniotic infection at term. J Infect Dis 145:1–8, 1982.
9. Altshuler G, Hyde S: Fusobacteria: An important cause of chorioamnionitis. Arch Pathol Lab Med 109:739–743, 1985.
10. Easterling TR, Garite TJ: *Fusobacterium:* Anaerobic occult amnionitis and premature labor. Obstet Gynecol 66:825–828, 1985.
11. Miller JM, Pupkin MJ, Hill GB: Bacterial colonization of amniotic fluid from intact fetal membranes. Am J Obstet Gynecol 136:796–804, 1980.
12. Romero R, Sirtori M, Oyarzun E: Infections and labor. V. Prevalence, microbiology, and clinical significance of intraamniotic infection in women with preterm labor and intact membranes. Am J Obstet Gynecol 161:817–824, 1989.
13. Goplerud CP, Ohm MJ, Galask RP: Aerobic and anaerobic flora of the cervix during pregnancy and the puerperium. Am J Obstet Gynecol 126:858, 1976.
14. Chow AW, Marshall JR, Guze LB: Anaerobic infections of the female genital tract: Prospects and perspectives. Obstet Gynecol Surv 30:477–494, 1975.
15. Gravett MG, Hummel D, Eschenbach DA, et al.: Preterm labor associated with subclinical amniotic fluid infection and with bacterial vaginosis. Obstet Gynecol 67:229–237, 1986.
16. Chaim W, Mazor M: Intraamniotic infections with fusobacteria. Arch Gynecol Obstet 251:1–7, 1992.
